# Around the Hospitals

**Published:** 1894-04-28

**Authors:** 


### AROUND THE HOSPITALS.

[Notice to the Managers of Institutions.—Special space is now reserved for the insertion of notices of hospital meetings and festivals. The Editor requests that all notices may reach The Hospital Office, 428, Strand, at least one week before the date of each meeting.]

**The Louth Charitable Infirmary.**—Several improvements have been made at this institution during the past year, which were specially subscribed for in a manner that shows a genuine interest in the institution. These improvements include sanitary annexes, much needed to place the Infirmary in a good hygienic condition. These were secured at a cost of £1,000, towards which the larger part has already been subscribed. A fancy dress ball, given by the staff, provided funds for the tiling and heating and other improvements in the operating theatre, and Miss Payne-Townsend has permanently endowed a bed, which she has hitherto maintained at a contribution of £30 per annum. Work at the Louth Charitable Infirmary has much increased of late years, especially in the direction of important operations. Larger subscriptions are required to meet the necessary expenditure, which exceeded the income by £56 during the past year. The number of patients received amounted to 1,012. Out-patients numbered 10,234.

**The Southport Convalescent Hospital.**—The management of the Southport Convalescent Hospital may well congratulate themselves on the prosperous condition of the institution, and the evident appreciation in which it is held. These two facts are proved by the balance to the good on the accounts, and the larger number of patients seeking admission. These numbered last year 3,710, being 135 in excess of any previous year. The Cotton District Convalescent Fund sent no less than 1,545 patients. Sea bathing is resorted to both in the hospital and on the shore. Last year the shore was much more resorted to than usually, and this fact caused a reduction in the hospital expenses. The improvements in the hospital during the past year consisted of new gas cooking stoves, and a range of steam cooking boilers. An excellent and kindly act on the part of the directors of the winter gardens and of the pier company has opened the gardens and pier of Southport to nurses of the institution free of charge. The income for 1893 amounted to £9,774, and the expenditure to £9,427. The cost per patient for the three weeks' stay in the hospital amounts to £1 12s. 2½d.

